# The Law of Lunacy and the Condition of the Insane in Scotland

**Published:** 1859-07-01

**Authors:** 


### Art. VI.—THE LAW OF LUNACY AND THE CONDITION OF THE INSANE IN SCOTLAND.

The first Annual Report of the Commissioners in Lunacy for Scotland has been recently published. The document is curiously instructive. Never was a necessity more thoroughly appreciated than that which led to the enactment of the Act "for the Regulation of the Care and Treatment of Lunatics, and for the Provision, Maintenance, and Regulation of Lunatic Asylums in Scotland," which received the Royal Assent on the 25th of August, 1857; never, it would appear, was an Act more ingeniously worded for rendering nugatory the presumed intentions of its framers; never was a Board created to carry out the intentions of an Act more impressed with the spirit, yet more perplexed with the letter of the law than the General Board of Commissioners in Lunacy for Scotland.

"The chief objects of the Statute," write the Commissioners, "are to provide for the building of district asylums for the reception of pauper lunatics, and to insure the proper care and treatment of lunatics generally, whether placed in asylums, or left in private houses under the care of relatives or strangers" (p. i.); but the provisions for attaining these ends are, in certain essential particulars, so peculiarly phrased that, if they were carried out to the letter, they would almost depopulate existing asylums, notwithstanding that one main object of the Act is to provide an increase of asylum accommodation. They would, also, remove a large number of pauper lunatics and lunatics under treatment in private dwellings from the control of the Commissioners, yet leave the latter responsible for their rightful care and treatment—the Act being, moreover, intended "to insure the proper care and treatment of lunatics generally;" and while permitting the Commissioners to have a wide range for "*suggestion*" (that pretty fiction by which the appearance of a reform is occasionally obtained without the reality), the provisions restrict their power of enforcing compliance with their suggestions in several of the most important matters relating to the care and welfare of lunatics.

The first Annual Report of the Commissioners is, indeed, valuable as showing the folly of legislating for the insane from a too strictly legal point of view; from the light it casts upon the difficulties, the dangers, and the eccentricities of Lunacy Legislation; and from much special information of wide and general application which it contains: and although the Commissioners state that, from the pressing character of their first year's duties, they have not been able to record the results of their experience so fully as they could have wished, yet the careful mode in which the Report has been drawn up deserves warm praise, and the excellent and conciliatory spirit displayed by the Commissioners amidst the difficulties with which they have had to contend must excite admiration.

We propose to examine the more generally interesting portions of the Commissioners' Report.

I. The *number* of lunatics in Scotland reported to the Commissioners on the 1st of January, 1858, and the manner of their distribution in asylums and private houses, were as follows:—

(1) In *Public Asylums*,—males, 1226; females, 1154; total, 2380. Of these numbers, 786 were private patients, and 1594 parochial paupers. In *Private Asylums*,—males, 330; females, 415; total 745. Of these numbers, 219 were private patients, and 526 parochial paupers. In *Poor-houses*,—males, 362; females, 487; total, 839. Of these numbers, 6 were private patients, and 833 parochial paupers. "This enumeration, however," the Report states, "cannot be considered accurate, as the Commissioners, in the course of their visits, found many paupers in poor-houses who had not been returned to us, but who were, nevertheless, persons of unsound mind." (p. ii.) (2) The Commissioners had not the means of obtaining any returns of the number of the insane in *Private Houses* possessing such claims to accuracy as would permit them to adopt them. The Statute confers no power upon the Commissioners to call for returns of private patients in the houses of relatives or others. "The number of the insane poor in private houses, as returned by the inspectors, amounted to 1784; of whom 810 were males, and 974 females. The subsequent investigations of the Visiting Commissioners, however, showed that the actual number of pauper lunatics was considerably greater, but to what extent will not fully appear until the returns for 1859 are received." (p. ii.)

The total number of the insane in Scotland on the 1st January, 1858, as returned to the Commissioners, and with the exception of private single patients, was 5748—*male*, 2718; *female*, 3030: *private*, 1011; *pauper*, 4737.

The proportion of pauper lunatics to paupers is thus stated by the Commissioners:—

"On 15th May, 1855, the number of registered paupers in Scotland amounted to 79,887: and the proportion of pauper lunatics to paupers, taking the number of the former at 3904, as reported by the Board of Supervision, was accordingly 4·88 per cent. On 15th May, 1857, the number of registered paupers amounted to 79,217, and the proportion of pauper lunatics to paupers, supposing the number of the latter to be the same on 1st January, 1858, as on 15th May, 1857, was thus 5·979 per cent. The basis on which this calculation is founded will appear erroneous if the number of registered paupers, as stated in the Twelfth Report of the Board of Supervision, be adopted as correct. They are there estimated at 69,217, or 10,000 below the actual numbers, a result apparently due to an error in addition. When this mistake has been corrected, the comments of the Board of Supervision upon the large decrease of paupers generally, and the nevertheless still increasing number of pauper lunatics, will be found to rest upon erroneous premises."—(p. iii.)

Steps have been taken by the Commissioners to ascertain as far as practicable the number of private insane, placed as single patients; but the investigation was not completed at the time of drawing up the report.

II. For the purposes of the Act, Scotland is divided into *eight* districts; but the Statute contains certain provisions for the alteration or modification of the districts, and these provisions have been so freely used that twenty-one districts have actually resulted. Indeed, in the initiatory arrangements for carrying out the objects of the Act, so far as districts were concerned, counties were permitted to exercise a somewhat wide discretion, without any efficient check as to the ultimate intentions of the Statute; and the result has been, not only that a multiplication of districts not contemplated has taken place, but also that different counties having given greater attention to local conveniences than to the intentions of the Act, the districts are, the Commissioners state—

"Very unequal as regards population, extent, and wealth; and several of them are perhaps too small to support efficient asylums. This remark is especially applicable to those which have been isolated, less perhaps by their own choice than from the refusal of the counties, with which their connexion seems more natural, to enter into combination with them. It is, however, not improbable that some modification of the existing arrangement may yet take place; but as the Statute confers no power to force one district into combination with another, the union of the isolated counties, with already constituted districts, is scarcely to be expected. Were any arrangements contemplated under the provisions of section 49, we might indeed stipulate for the reception of isolated counties into the new district before approving of the proposed combination, but there is little prospect of any application being made to us to alter or vary the districts as at present constituted."—(p. vi.)

III. The districts having been formed, the Commissioners had to make the best they could of them. And, first, it was necessary to determine "whether the existing accommodation for the insane poor in each district was sufficient for its wants; or, whether, and to what extent, additional accommodation should be provided." Here, however, a serious hitch in the phraseology of the Statute was experienced; for it was very doubtful what the meaning of the term "existing accommodation," as used in the Act, was. There was no lack of statutory explication; but, unfortunately, it did not very well apply to the object it was intended to elucidate—existing accommodation *in Scotland*. It was doubtful whether the Commissioners were called upon to recognise private asylums and the lunatic wards of poor-houses: it was questionable whether District Boards were not compelled to adopt the accommodation aforesaid, and use it for the reception of the pauper lunatics of the district before proceeding to erect a district asylum: and it was certain that local authorities asserted that the lunatic wards of poor-houses not only constituted existing accommodation, but were, in fact, public asylums. Counsel were called in to explain the explanatory clauses of the Statute, and to set the matter at rest in so far as the lunatic wards of poor-houses were concerned; and the said counsel "advised that poor-houses, including their lunatic wards, could not be recognised or licensed in terms of the Act"—thus confirming the very proper opinion of the Commissioners that they *ought* not to be so recognised. Parliament was, however, invoked, and on the 2nd August, 1858, a short *amendment* was passed, by which the Commissioners were empowered to grant licenses for the reception of pauper lunatics into wards of poor-houses, for a period of five years, from 1st January, 1858, "as it is expedient that provision should be made for the custody of pauper lunatics till district asylums are ready for their reception." One parochial board, however, that of Barony, unawed by the Commissioners, by counsel, and by Parliament, exhibits a tenacity of parochial rights befitting the hey-day of local authority, and has intimated that it considers the lunatic wards of its poor-house as embraced by the statutory definition of a public asylum. Although the parochial board has been compelled to apply for the Commissioners' license, it has done so on the stipulation that this step shall not be held as implying an abandonment of the claim. The Edinburgh City poor-house also holds out on the ground that its lunatic ward represents the Old City Bedlam.

Having defined the meaning to be attached to the term "existing accommodation," and stated the mode in which this meaning was arrived at, the Commissioners proceed to report upon the amount of existing provision for the insane poor in the various districts as now constituted, and to describe the measures which are in progress for supplying such deficiencies as, on investigation, have been ascertained. The details under this head are preceded by sundry remarks of considerable interest upon the objects which should be had in view in the admission of lunatics into asylums. The Commissioners write—

"We do not conceal from ourselves the practical difficulties which lie in the way of determining with accuracy the number of insane at large who should be placed in asylums. The conclusions at which we arrived were not altogether based on the nature or curability of the malady, but were influenced also by the circumstances in which the patient was placed, and the degree of care bestowed upon him. We asked ourselves, whether in the interests of the patient himself, or in those of society, it seemed most desirable to place him in an asylum or to leave him at home; and our decision was taken upon a general consideration of all the facts of each case. For, in addition to the mental and bodily condition of the patient, as well as the general circumstances by which he was surrounded, we felt bound also to take into account the constitution of our asylums; and we were conscious that our difficulties would often have been materially lessened had these establishments been based on the idea of providing a diversity of accommodation for patients affected with different degrees of mental incapacity. There are many persons, for example, whose mental condition requires that they should be placed under the care and control of others, yet whom we would hesitate to deprive of liberty to the extent almost necessarily involved in sending them to lunatic asylums as at present constituted.

"There is a growing conviction throughout Europe, manifested in the writings of various psychological writers of repute, in England, France, Germany, Belgium, and even Spain, that the constitution of lunatic asylums requires great modification. This opinion is founded chiefly on the diversity of the forms of insanity; but it rests also on the difficulty of suitably providing for the always increasing number of the insane. It may be open to question, whether this difficulty is caused by an actual increase of persons affected with insanity, or whether it is simply due to an accumulation of the insane through the prolongation of their lives by better care. It is not unlikely that both causes may be in operation; but, at the same time, it is probable that the increase is in a great degree only apparent, arising from more attention being directed to the subject of insanity, and the consequent discovery of a greater number of persons affected with the malady. However, be this as it may, it is an ascertained fact, that the erection of an asylum always increases the known numbers of lunatics, by bringing into public notice cases which were formerly hidden from view in private houses. Beyond all question, transference to an asylum is very generally calculated to prove most beneficial to an insane patient, but the extent to which asylums have contributed to diminished insanity is not so easily determined. Statistical returns, it is true, show that, under present circumstances, nearly one half of the cases admitted into these establishments are restored to sanity; but at the same time it must be remembered that we have no means of forming an opinion as to what the result would have been had the treatment of these patients been conducted in private houses.

"The question, however, is one of great practical importance, because on its answer naturally hinges a powerful argument in favour of or against the extension of asylums. But we must here state our conviction that the influence of asylums in restoring patients to sanity cannot be fairly tested by the experience of the past, for hitherto their curative agency has to a very considerable extent been neutralized by the combined effect of neglect, prejudice, and ignorance. It cannot be too often repeated, that in the treatment of insanity, loss of time is unfavourable to recovery, and that every impediment that is thrown in the way of immediate treatment acts most prejudicially upon the patient by tending to render permanent the aberration from normal action, which, under favourable circumstances, would speedily have subsided. We are, therefore, of opinion, that asylums are capable of rendering to humanity far greater services than they have yet achieved. There cannot, however, be the smallest doubt, that these establishments have, even in times past, proved of great public utility, by undertaking the treatment and management of patients requiring special medical care, and of those whom, from violence or other peculiarity, it is found dangerous or impossible to retain in private houses. Moreover, it has been clearly proved that the discipline of an asylum exercises a most beneficial and curative influence upon many patients who, if left at home, would probably have become confirmed lunatics, and is calculated to ameliorate in a very remarkable manner the condition even of the most intractable incurable cases. It is very certain, then, that asylums prove of the greatest service both to the patients and the public; and, therefore, the question to be considered is, not whether their extension is required, but whether, as at present constituted, they fulfil all the expectations which led to their erection, and which the expense of their maintenance might warrant us in entertaining. Beyond all other aims, an asylum should have for its object the cure of the insane and the diminution of insanity. Now, in relation to this malady, two important facts have been clearly established, first, that one chief cause of the affection is hereditary predisposition; and secondly, that the success of curative treatment depends in a very great degree upon its being undertaken at an early stage of the disease. In the course of our investigations, we have obtained abundant proof that fatuous female paupers frequently become the mothers of illegitimate children, who, in their turn, grow up imbeciles, or become lunatics; and although there is naturally more difficulty in tracing the source of idiotcy or insanity to a paternal origin, there can be little doubt that male fatuous paupers contribute to this evil. In illustration of these remarks, we shall here give the result of our investigations in one county into this painful aspect of insanity. The number of single patients visited or reported on amounted to 349. Of these, 33 were reported to the visiting Commissioners as illegitimate; 22 being registered paupers, and the remaining 11 indigent private cases.

Of the 349, 113 were females above 17 years of age. Of these, 22 were in circumstances affording adequate protection to their chastity. Of the remaining 91, 15 were known to have given birth to illegitimate children, and 5 to have borne more than one child. Of the 15 mothers, 3 are known to have been illegitimate, and 12 are at present paupers; of their children, 6 are known to be idiots. There are, besides, in the county, 3 other idiots who are known to be the offspring of insane or imbecile mothers, who are dead or have disappeared. These facts are most deplorable; nevertheless, it would be esteemed a harsh measure to send all such cases to asylums, and yet society has a right to demand that all persons who are supported on charitable funds should be placed in such circumstances, and under such control, as will guard against the propagation of this social evil. This result, we are of opinion, might be obtained by attaching to asylums adjunct houses, in which such patients, and others of analogous character, could be placed, without to the same extent depriving them of liberty as the patients in the asylum proper. And we are further of opinion, that many of the objections at present entertained, both by the friends of such patients and the public generally, in regard to placing them in asylums, would be obviated by the proposed modification of these establishments. Moreover, experience shows that there is frequently great unwillingness on the part of relatives to send to asylums patients who are suffering from the milder and incipient forms of insanity. Yet these are precisely the cases in which removal from the home circle is most likely to exercise a beneficial influence. This unwillingness appears to be in a great measure due to the necessity of obtaining two medical certificates of insanity and the sheriff's order, before a patient can be placed under treatment—formalities from which many sensitive minds shrink until the malady has become confirmed. Indeed, it may be said that these precautions, which are intended for the welfare and protection of the patient, are frequently calculated to affect him most injuriously, by delaying appropriate treatment until the mental aberration has become so apparent, that two medical men, on a cursory examination, can, without hesitation, certify to its existence. On this account, we are inclined to think that adjunct houses, in which patients, affected with certain forms of insanity, could be received without the strict legal formalities at present required, would prove a beneficial modification of our asylums, and would tend to increase recoveries, by inducing patients and their friends to have recourse to treatment before the malady had become confirmed."—(pp. ix.—xi.)

The truthfulness of the foregoing remarks may be fully conceded, and the last suggestion contained in them merits careful consideration from its importance.

The illustration given of the propagation of idiotcy or insanity by the child-bearing of fatuous females, although sufficiently painful in the brief summary contained in the above quotation, becomes still more so by a reference to the Appendix of the Report, a section of which, entitled *Illegitimacy and Erotic Tendencies* (p. 195), contains a brief account of the several instances alluded to by the Commissioners, than which a more painful picture of degradation or one requiring more prompt and decisive measures of interference cannot well be conceived.

The amount of asylum accommodation existing in, or which can be had recourse to by, different districts varies greatly, and it is not easy to estimate the amount of deficiency in each case. The Commissioners detail at length the circumstances of each district, and the following illustration may be perhaps received as setting forth the *plus* and *minus* degrees of deficiency.

"If we take the counties of Forfar, Edinburgh, and Lanark, as examples of districts already tolerably provided for in this respect, we find that of the 1617 pauper lunatics with which they are chargeable, 1360 or 84·11 per cent. are in asylums or poor-houses, and that only 257 or 15·89 per cent. are left at home. Whereas, if we take the counties of Caithness, Ross and Cromarty, Sutherland, and Inverness, which are altogether unprovided with asylum accommodation, we find that of the 492 pauper lunatics with which they are chargeable, only 136 or 27·64 per cent. are in asylums, and no less than 356 or 72·36 per cent. are left at home. Taking, accordingly, the first set of counties as a standard, it follows, that in the northern counties there are at least 277 pauper lunatics at home who should be removed to asylums."—(p. xii.)

IV. But the Commissioners were not only placed in tribulation by the vagueness and insufficiency of the statutory definition of "existing accommodation;" they were also, and still are, reduced to great straits by the definition of the word *lunatic*, which the statute declares "shall mean and include any mad or furious or fatuous person or persons so diseased or affected in mind, as to render him unfit in the opinion of competent medical persons to be at large, either as regards his own personal safety and conduct or the safety of the persons and property of others or of the public." Whether we regard this explication as a piece of composition, or in reference to the presumed ultimate intentions of the Act, we are equally lost in wonder. If we note the former, we cannot but admire the vagueness of the definition; for, had the sentence been penned with the especial object of leading to a contention of opinion, the point could not have been more aptly hit: if we note the latter, nothing can be imagined better calculated not simply to cause difficulties in the execution of the Act, but, if the definition were followed to its most legitimate conclusion (if any one conclusion more legitimate than another may be supposed to arise out of or may be derived from it) to render the whole Act almost useless.

"The question here arises, [write the Commissioners,] whether the second part of the definition is simply explanatory of the first part, or whether it is an amplification of the definition; whether, namely, every mad, fatuous, or furious person is *simpliciter* a lunatic; or whether to be so accounted he must *also* be unfit to be at large, as regards his own safety and conduct, or the safety and property of the public. It may further be considered doubtful, whether it is contemplated that a person, in order to be declared a lunatic, must be unfit to be at large as regards *both* his own safety and conduct, or as regards *both* the safety and property of the public; or whether the definition will be fulfilled if he be unfit to be at large, as regards *either* his own safety or conduct, or as regards *either* the safety or property of the public. In practice, the view has generally been adopted, that every person certified to be of unsound mind is, in the statutory sense, a lunatic; but the Board of Supervision appear to be of opinion that no pauper of unsound mind can be considered a lunatic in terms of the Statute, unless there is also reason to apprehend danger. (See Correspondence in Appendix D.) In accordance with this view, it has on various occasions been maintained not only by parochial boards, but also by sheriffs, that fatuous or idiotic paupers, although totally incapable from mental deficiency of acting for themselves, are not lunatics in terms of the Act. The question, therefore, is one of great practical importance, and its early adjustment is extremely desirable."—(p. xxvi.)

The correspondence referred to in the foregoing quotation forms a very interesting episode illustrative of the contention between the *spirit* and the *letter* of the law.

The vagueness of the statutory definition of the term "lunatic" constitutes a ready resting point in the majority of instances, where it may be an object to throw obstacles in the way of the Commissioners carrying out the Act. The other difficulties with which they have had mainly to contend in the disposal of lunatics, have been the want of asylum accommodation; the mistaken affection of relatives; the penuriousness of parochial boards; and certain objections made by medical men to grant certificates of lunacy. The last-mentioned difficulty has not been of very frequent occurrence, but it is of considerable importance. The Commissioners state that—

"By the Statute, all pauper lunatics shall be sent to the asylum of the district in which the parish of the pauper is situated, unless the board agree to their disposal otherwise. But the statutory form of the medical certificate of insanity required to place a patient in an asylum, includes an expression of opinion that he is a proper person to be detained under care and treatment; and, accordingly, some medical men, while admitting a patient to be of unsound mind, have refused to certify that he was a proper person to be detained under care and treatment, when the question of sending him to an asylum was also involved. The practical result of such refusal is to deprive the board of all power to compel improvement in the condition of a patient; and, in this way, a pauper lunatic, for whose care we are legally responsible, if not certified 'to be a proper person to be detained under care and treatment,' is practically removed from our jurisdiction, without being placed under that of the Board of Supervision, whose authority, in matters of treatment, is now limited to ordinary paupers.

"Occasionally, also, medical men have refused to grant certificates, in the cases of patients suffering under certain forms of insanity, on the ground that they do not come within the scope of the Act.

"From the vague and unsatisfactory nature of the definition of lunacy, we have, in several instances, had no alternative, but with regret to yield our own views to those expressed by the local medical men, and to leave the patient in circumstances which we considered unsuitable."—(p. xxx.)

Another important difficulty in the effective carrying out of the objects of the Statute has proved to be the provision, that in no case shall a lunatic be admitted into an asylum without an order from the sheriff. This vexatiously impedes the early reception of patients into asylums without any resulting benefit of importance. The Commissioners write—

"In granting his order for the detention of a lunatic, the sheriff exercises judicial functions; for he takes into consideration the terms of the medical certificates, and grants or withholds his order according as he considers the facts therein stated as sufficient proof or otherwise of insanity in a statutory sense. Accordingly, it frequently happens that the sheriff refuses to accept the medical certificates as sufficient warrant for him to grant his order; and on one occasion, on which the patient had been received on a certificate of emergency, this refusal was necessarily followed by immediate discharge. This difference of opinion between the sheriff and the medical men occurs principally in regard to dipsomania, as a patient affected with this form of insanity is not considered by some sheriffs as a lunatic in terms of the Statute. But the cause of the refusal of the sheriff to grant his order lies sometimes merely in the fact, that the circumstances stated in the medical certificates, as indicating insanity, do not in his opinion afford sufficient evidence of its existence. In this respect, however, great differences occur in practice, as some sheriffs reject statements which others consider sufficient; while others receive, as satisfactory, statements which certainly do not meet the requirements of the Statute."—(p. xxxiii.)

The Commissioners suggest certain changes by which the objections that at present attach to the sheriff's order may be done away with, and still its value remain as a "guarantee against the granting of hasty or improper certificates of insanity by medical men."

It is necessary to remark that difficulties are occasionally thrown in the way of the sheriff's coming to a right conclusion upon the cases submitted to him, from the careless mode in which the medical certificates are sometimes filled up.

The Commissioners next comment upon the powers which they possess in respect to the visitation of asylums, and the insufficiency of the means as yet at their command, from the imperfect carrying out of the statute by district boards, for the efficient inspection of the whole of Scotland. They also suggest that district asylums, in order that local obstacles be avoided, should be supported by a general district rate, or a rate of the whole country, and not by parochial rates, as set forth by the present law—each parish supporting directly the burden of its pauper lunatics. The defective provisions of the statute for the discharge of patients from asylums form, moreover, a subject of remark in the Report.

Concerning the condition of lunatics placed singly in private houses, the Commissioners report of those who are paupers, that although in several districts their state is most miserable, yet that a large proportion of the cases "are treated with kindness and consideration." "On this fact," the Commissioners observe,—

"Rests our chief hope of the success of the cottage system of accommodation, should it be considered proper by district boards to give it a trial as an adjunct to their district asylums. For, if kind and humane treatment be extensively found in cottages, even under the present system of imperfect supervision, there is every reason to think that, under the immediate superintendence of the asylum officers, it could be so fostered in growth as to open up a prospect of escape from the many questions that are every year rendering the care and management of the insane poor a problem of more difficult solution. In every country of Europe, the question of the accommodation of the insane is daily becoming more and more embarrassing; and we see how in England, notwithstanding the wealth of the country, and the humane spirit of the people and of the Legislature, the increase in the number of lunatics keeps ahead of all the exertions made for their accommodation. This is a grave fact which deserves our most serious consideration before we commit ourselves to the building of asylums, in the expectation that no further call will be made upon us. No doubt, it is theoretically easy to maintain the doctrine that asylum accommodation should be provided for all the insane poor, and that no expense should be spared in supplying the wants of this afflicted class. But the sane poor have also their claims; and the question may be asked, how far it is right, that an idiot, or a lunatic in a state of dementia or general paralysis, who is beyond all hope of being restored to sanity; and who, moreover, is little able to appreciate kindness, or to derive pleasure from the care and attention bestowed upon him, should receive treatment greatly superior to that bestowed upon an aged or infirm ordinary pauper, who, though in a sense also incurable, is more capable of appreciating kindness and showing gratitude in return? In England, the poor-house is open to the able-bodied labourer, but in Scotland it is reserved for the aged and helpless poor; and, accordingly, with us there is not perhaps the same reason for drawing a distinction between the treatment of ordinary paupers and that of incurable pauper lunatics. But there will always be this essential difference between the two classes, calling for special consideration in their treatment, that the latter are labouring under a degree of mental incapacity which renders them altogether dependent upon the care of others, and incapable of appealing against harshness or neglect. Still, as we must place a limit on our charitable expenditure, we should beware of making such a distinction in their treatment as might raise a doubt as to its propriety; and must therefore take care not to be too lavish with the one hand, lest we be forced to be too penurious with the other. On this account, we lean towards any scheme that will embrace good and economical accommodation for the whole insane poor, rather than to one which, from the expense of carrying it out, will sooner or later be of only partial application."—(p. li.)

From the returns given by the Commissioners, it seems that, on the 1st January, 1858, the number of pauper lunatics amounted to 4737, of whom 2120 were in asylums and licensed houses, 833 in poor-houses, and 1784 in private houses. The average weekly allowance for out-door relief amounted in the districts from which returns of the allowance had been made, to 2*s.* 10¼*d.* This, however, on account of a few exceptional cases of allowance, would be rather above the mark; but, it must be added, that grants of clothing are also made.

The Commissioners speak highly of the general condition of the patients in the public asylums. They remark:—"In none of the asylums have we observed mechanical restraint in use; and the registers show that it has been resorted to in one or two instances only, in which there appeared to be good grounds for its application. Seclusion for short periods is in frequent use; but no case has come under our observation or notice, in which it has been improperly applied or injuriously extended" (p. liv.). They add also:—"The extent to which amusement and recreation have been carried is a remarkable and very gratifying feature in most of the asylums; but in all of them there is still a deficiency of objects calculated to arrest the attention and rouse the feelings of the patients. We, however, notice with pleasure a gradual improvement in this respect, which is more especially apparent at Glasgow" (p. lv.).

Particular attention is directed to the wretched and very insufficient stipends of the assistant medical officers in the different asylums; and the Commissioners express a regret that in none of the asylums measures have been taken to secure the superintendents retiring allowances.

The average number of patients resident in the public asylums in 1858 amounted to 2421, of whom 1253·5 were males, and 1167·5 females. The admissions, were 449 males and 498 females; recoveries, 151 males and 201 females; discharges not recovered (many being transferred to other asylums), 149 males, and 140 females; deaths, 109 males, and 94 females. Proportion of recoveries per cent. on admissions, 33·630 males, and 40·361 females; proportion of deaths per cent. on numbers resident, 8·699 males, 8·051 females.

Of the condition of the pauper patients in licensed houses, the

Commissioners speak somewhat unfavourably, although considerable improvements have taken place since the Report of the Royal Commissioners in 1857, and many improvements are still being effected. Of the pauper licensed houses it is stated that they "are generally overcrowded" (mainly from the inadequacy of the accommodation for the insane throughout the country generally),—

"Though the evil, from the precautions we have taken, is gradually diminishing. The ventilation, especially during the night, is in a corresponding degree imperfect. The rooms, however, are generally comfortably heated, and the furniture has, to a certain extent, been improved by supplying tables, and benches with backs; but the system under which these houses exist is fundamentally wrong, and there is, and must continue to be, a great and pervading want of cheerfulness and amenity.

"The patients, when within doors, are generally found sitting in cheerless rooms, ranged on benches, listless, and without occupation; and, when out of doors, they are usually lounging sluggishly about the airing-courts, or are crouching in corners. But we have pleasure in stating that, in regard to Millholm House and Longdale, this description admits of considerable modification, as in both of these establishments praiseworthy exertions have been made to provide occupation for the patients. At Longdale, with 130 patients, Dr. Muirhead has about eighty acres of land in possession, the produce of which is all consumed on the premises. The house is supplied with milk and butter from his own cows; he feeds his own stock; raises his own vegetables; and evidently turns these farming operations to good account. His experience in this respect should be received as a valuable hint by the district boards, as it tends strongly to show that a good-sized farm ought to be an economical appendage to an asylum.

"The diet and clothing of the patients have generally been found sufficient, and there has accordingly been no recurrence of that excessive mortality, the result of cold and starvation, which called for such severe comments from the Royal Commissioners. We have, however, still reason to doubt, principally from the low condition of the vital powers of the patients, whether the diet was always appropriate; and in one instance we have pointedly directed the attention of the medical attendant to the subject. There seemed to us to be a want of sufficient variety in the food, and possibly also an insufficiency of nutritive principles. Our views on this head were confirmed by the improvement in the physical condition of the patients which followed on a change of diet. The error here committed was due to the ignorance of the proprietor, who did not seem to be aware of the necessity of varying the food; and this fact alone is sufficient to show the impolicy of confiding the care of even incurable patients to uneducated men. We take this opportunity to state, that in the only instance in which we have granted our license to a new proprietor, the licentiate had received a professional education.

"Mechanical restraint has been almost entirely banished from the licensed houses, and patients who are recorded in the Report of the Royal Commission as almost always under restraint, are now habitually freed from their bonds."—(pp. lix.—lx.)

The average number of insane resident in the licensed houses in 1858 amounted to 817, of whom 355 were males and 462 females. The admissions, were 125 males and 222 females; the recoveries, 48 males, 86 females; discharges not recovered (several being transfers to other asylums), 21 males, 35 females; deaths, 30 males, 35 females. The proportion of recoveries per cent. on admissions was 38·400

males, 38·738 females; the proportion of deaths per cent. on the numbers resident, 8·450 males, 7·575 females.

Concerning the two idiot schools which exist in Scotland, the Commissioners express the conviction that, as at present conducted, they will not prove ultimately successful. In the one school the training is too scholastic; in the other, although sounder principles of tuition prevail, the funds are unfortunately too scanty to permit of their being fully carried out. The Commissioners insist that the preliminary training of idiots should be chiefly physical, and that in tutoring the senses objects should be had recourse to, not books. Above all, in the more educable idiots the training should mainly be in useful physical occupations.

The treatment and accommodation of lunatics in poor-houses is emphatically and most righteously condemned in the Report. The average number of insane resident in poor-houses in 1858 was 746, of whom 131 were males and 201 females; the admissions were, 131 males and 201 females; the recoveries, 45 males, 92 females; discharges not recovered, 28 males and 41 females; deaths, 49 males, 53 females; proportion of recoveries per cent. of admissions, 34·351 males, 45·771 females; proportion of deaths per cent. on numbers resident, 15·909 males, 2·087 females.

The Commissioners point out the imperfections and inconsistencies of the provisions respecting dangerous lunatics and the evils resulting therefrom, as well as the harsh and oppressive manner in which the regulations respecting alien lunatics are carried out. Further, certain suggestions respecting the management of criminal lunatics are given, and, after expressing an opinion against the establishment of an especial asylum for lunatics of this class, the Commissioners propose that "special wards for their reception should be provided in connexion with one of the district asylums. The chief advantage of this scheme would be the supervision and management of the patients by an experienced medical superintendent and skilled attendants" (p. lxxviii.). The whole of the criminal lunatics in Scotland do not, on an average, exceed thirty, and there is not much probability that this number will ever be greatly increased.

The Report concludes with a statement of the powers which the Commissioners possess respecting the property of lunatics. The Statute sets forth that the General Board shall perform sundry important duties protective of the property of a lunatic; but the means provided and the powers granted for the performance of these duties are so limited that the intentions and provisions of the Act are practically nullified (p. lxxix.).

It is to be hoped that the shortcomings in phraseology, drawing-up, and working of the New Scotch Lunacy Act, will operate as a caution to those upon whom the duty may fall of amending or remodelling the English and Irish Lunacy Acts; and we would recommend all who are interested in lunacy legislation to read carefully the First Annual Report of the Scotch Commissioners in Lunacy.

